# Comprehensive analysis of gut microbiota of a healthy population and covariates affecting microbial variation in two large Japanese cohorts

**DOI:** 10.1186/s12866-021-02215-0

**Published:** 2021-05-20

**Authors:** Jonguk Park, Kumiko Kato, Haruka Murakami, Koji Hosomi, Kumpei Tanisawa, Takashi Nakagata, Harumi Ohno, Kana Konishi, Hitoshi Kawashima, Yi-An Chen, Attayeb Mohsen, Jin-zhong Xiao, Toshitaka Odamaki, Jun Kunisawa, Kenji Mizuguchi, Motohiko Miyachi

**Affiliations:** 1 Laboratory of Bioinformatics, Artificial Intelligence Center for Health and Biomedical Research, National Institutes of Biomedical Innovation, Health and Nutrition, Osaka, 567-0085 Japan; 2 Morinaga Milk Industry Co., Ltd., Next Generation Science Institute, Kanagawa, 252-8583 Japan; 3 Department of Physical Activity Research, National Institutes of Biomedical Innovation, Health and Nutrition, Tokyo, 162-8636 Japan; 4 Laboratory of Vaccine Materials, Center for Vaccine and Adjuvant Research and Laboratory of Gut Environmental System, National Institutes of Biomedical Innovation, Health and Nutrition, Osaka, 567-0085 Japan; 5 Faculty of Sport Sciences, Waseda University, Saitama, 359-1192 Japan; 6 Faculty of Human Nutrition, Tokyo Kasei Gakuin University, Tokyo, 102-8341 Japan; 7 Faculty of Food and Nutritional Sciences, Toyo University, Gunma, 374-0193 Japan

**Keywords:** 16S rRNA, Gut microbiota, Covariates, Large cohort, Japanese population

## Abstract

**Background:**

Inter-individual variations in gut microbiota composition are observed even among healthy populations. The gut microbiota may exhibit a unique composition depending on the country of origin and race of individuals. To comprehensively understand the link between healthy gut microbiota and host state, it is beneficial to conduct large-scale cohort studies. The aim of the present study was to elucidate the integrated and non-redundant factors associated with gut microbiota composition within the Japanese population by 16S rRNA sequencing of fecal samples and questionnaire-based covariate analysis.

**Results:**

A total of 1596 healthy Japanese individuals participated in this study via two independent cohorts, NIBIOHN cohort (*n*=954) and MORINAGA cohort (*n*=642). Gut microbiota composition was described and the interaction of these microorganisms with metadata parameters such as anthropometric measurements, bowel habits, medical history, and lifestyle were obtained. Thirteen genera, including *Alistipes*, *Anaerostipes*, *Bacteroides*, *Bifidobacterium*, *Blautia*, *Eubacterium halli* group, *Faecalibacterium*, *Fusicatenibacter*, *Lachnoclostridium*, *Parabacteroides*, *Prevotella_9*, *Roseburia,* and *Subdoligranulum* were predominant among the two cohorts. On the basis of univariate analysis for overall microbiome variation, 18 matching variables exhibited significant association in both cohorts. A stepwise redundancy analysis revealed that there were four common covariates, Bristol Stool Scale (BSS) scores, gender, age, and defecation frequency, displaying non-redundant association with gut microbial variance.

**Conclusions:**

We conducted a comprehensive analysis of gut microbiota in healthy Japanese individuals, based on two independent cohorts, and obtained reliable evidence that questionnaire-based covariates such as frequency of bowel movement and specific dietary habit affects the microbial composition of the gut. To our knowledge, this was the first study to investigate integrated and non-redundant factors associated with gut microbiota among Japanese populations.

**Supplementary Information:**

The online version contains supplementary material available at 10.1186/s12866-021-02215-0.

## Background

Trillions of microorganisms constitute the gut microbiota in the human gut; it is a complex ecosystem and is directly or indirectly related to intestinal [1] and metabolic [2, 3] diseases, and neurological disorders [4, 5]. Owing to inter-individual variations in the gut microbiota composition among healthy populations [6], it is important to comprehend the characteristics of healthy gut microbiota and its determinants for the maintenance of human health and well-being [7]. Major host factors that have been reported to affect the gut microbial community structure include: age [8,10], ethnicity [11, 12], geography [13], host genetics [14], gender [15], and life style factors such as dietary habits [16], smoking [17], exercise [18], and medications of non-antibiotics [19] and antibiotics [20]. Certain patterns and transitions in the gut microbiota composition are observed with age [8] and altered dietary intake [21,23]. Furthermore, intestinal transit time, which is measured directly or assessed using Bristol Stool Scale (BSS) scores [24], is also a factor influencing the composition and diversity of gut microbiota [25, 26]. The association between intestinal transit time and gut microbiota is complicated and interactive [27, 28], and therefore, studies involving the analysis of transit time as one of the investigating factors will help us to improve our understanding of the gut microbiome.

Several reports have investigated the relationship between gut microbiota and variable factors, however, most of those reports identified only a few factors without integration and non-redundant estimation of host and environmental impact. In 2016, Falony et al. [29] and Zhernakova et al. [30] reported the estimation of variable factors in Belgian and Dutch cohorts. However, those studies only compared results separately obtained from two independent cohorts and ignored the disagreement of some variable factors among different cohorts. Significant effects of determinants such as age, BSS score, and body mass index (BMI) on gut microbiota were analyzed in two independent cohorts in the study by Falony et al. [29]. These determinants are reported to considerably affect not only European populations, but also cause gut microbiota variations in Chinese populations [31].

The gut microbiota may exhibit a unique composition depending upon the country of origin and race of the individual [32]. To comprehensively understand the link between healthy gut microbiota and host state, it is beneficial to conduct large-scale cohort studies. A previous study has confirmed that the gut microbiome of Japanese populations exhibits a specific composition [32], different from that of other populations. Three previous reports [8, 33, 34] have described determinants of healthy gut microbiota in Japanese cohorts and analyzed the relationship of gut microbiota with these determinants in more than one hundred subjects, however, the scale and metagenomic data analysis were comparatively limited [29, 30]. Thus, an integrated view of the non-redundant factors affecting gut microbiota composition among the Japanese population is yet to be established.

In this study, we analyzed gut microbiota composition, including the structure and interaction of gut microorganisms with metadata parameters such as anthropometric measurements, bowel habits, medical history, and lifestyles of Japanese populations. We obtained data from two independent cohorts, NIBIOHN cohort (v20.1) and MORINAGA cohort (v20.1) including 954 and 642 Japanese individuals, respectively. Moreover, we have not only compared the statistical results from our two cohorts but also unified the sets of phenotypic parameters used in the two studies as much as possible. Considering the batch effect between the two large cohorts, the analysis results of each cohort were compared without integration. The comparative data analysis between the two large distinct cohorts improved the robustness and reliability of our results. This is the first report to identify the non-redundant association of host factors with gut microbiota among Japanese populations.

## Methods

### Study population

We evaluated fecal samples and metadata of community-dwelling Japanese volunteers from two independent cohorts: health and nutrition-based cohort study conducted by National Institutes of Biomedical Innovation, Health and Nutrition (NIBIOHN), hereafter referred to as NIBIOHN cohort, and FAECES-02 study conducted by Morinaga Milk Industry, hereafter referred to as MORINAGA cohort.

In the NIBIOHN cohort, a total of 954 healthy Japanese adult volunteers were analyzed from October 2015 to June 2019 (age range of 2080, male: 391, female: 563). In the MORINAGA cohort, a total of 642 healthy Japanese adult volunteers were analyzed from March 2018 to May 2019 (age range of 2183, male: 129, female: 513). There were some differences in the characteristics of the two cohorts, in particular the distribution of residential area of the participants was extremely different; NIBIOHN cohort comprised of individuals from a limited area with a high concentration of subjects in each area, and MORINAGA cohort comprised of individuals from a dispersed and continuous area (Fig. S1). Most of the participants in the MORINAGA cohort were customers who purchased products of the Morinaga Milk Industry Co., Ltd. including dairy products or probiotics supplement. All participants in both cohorts were physically independent, did not have a history of cancer, cardiovascular, liver or gastrointestinal disease, and no health problems requiring attention were detected after close examination. In addition, candidates who took antibiotics, laxatives, or anti-inflammatory drugs up to 2weeks prior to the study and whose data quality did not meet the criteria described below were excluded. Signed informed consent was obtained from all participants. These cohort studies were approved by the Ethics Committee of National Institutes of Biomedical Innovation, Health and Nutrition (Osaka, Japan), and by the Ethics Committee of Japan Conference of Clinical Research (Tokyo, Japan) and adhered to all guidelines.

### Questionnaire about covariates

All participants answered a questionnaire on anthropometric data, medical history, fecal characteristics, dietary habits, physical activity, and sleep; a total of 76 items common to two cohorts were extracted as variables (Table S1). Fecal volume, form and color were evaluated by using our assessment card tool for NIBIOHN cohort as described previously [35] with slight modifications related to volume and color for MORINAGA cohort. To assess daily dietary intake, we asked subjects to answer a different questionnaire, the Brief Self-Administered Diet History Questionnaire (BDHQ), to estimate the intake amount of 58 food and beverage items consumed in the preceding month [36] (Table S1). The subjects whose estimated total energy intake was under 600kcal or over 4000kcal were excluded due to lack of data reliability.

### Fecal sampling

Fecal samples from both cohorts were self-collected at home without water immersion using a fecal catcher. A red bean-sized fecal sample was collected, and an aliquot was immediately and completely mixed with 3ml of guanidine thiocyanate solution (TechnoSuruga Laboratory, Shizuoka, Japan) prior to its transport to the laboratory at room temperature.

### DNA extraction and 16S rRNA gene amplicon sequencing

Fecal sample mixtures were mechanically disrupted by the bead beating method using 0.1mm glass beads, DNA was extracted using an automated extraction machine, and the V3-V4 region of the bacterial 16S rRNA gene was amplified by PCR. All samples from both cohorts were processed similarly, with some modifications as mentioned below.

For NIBIOHN cohort, the fecal sample mixtures were mechanically disrupted using Cell Destroyer PS1000 (Bio Medical Science, Tokyo, Japan). DNA was extracted by using Gene Prep Star PI-80X device (Kurabo Industries, Osaka, Japan). The V3-V4 region of the 16S rRNA gene was amplified using KOD-Plus-v2 (Toyobo, Osaka, Japan) and sequenced by paired-end method using Illumina MiSeq instrument and the MiSeq v3 Reagent Kit (Illumina, San Diego, CA, United States). The V3V4 region of the 16S rRNA gene was amplified using the following primers: forward, 5-TCGTCGGCAGCGTCAGATGTGTATAAGCGACAGCCTACGGGNGGCWGCAG-3, and reverse, 5-GTCTCGTGGGCTCGGAGATGTGTATAAGAGACAGGACTACHVGGGTATCTAATCC-3. All the steps from fecal sampling to 16S rRNA gene amplicon sequencing was performed according to a previously described protocol [37].

For MORINAGA cohort, the fecal sample mixture was mechanically disrupted using FastPrep-24 5G (MP Biomedicals, Santa Ana, CA, United States). DNA was extracted by using Gene Prep Star PI-480 device (Kurabo Industries). The V3-V4 region of the 16S rRNA gene was amplified using TaKaRa Ex Taq HS Kit (Takara Bio, Kusatsu, Japan) and sequenced by paired-end method using Illumina MiSeq instrument and the MiSeq v3 Reagent Kit (Illumina). The V3V4 region of the 16S rRNA gene was amplified using the following primers: forward: 5- CGCTCTTCCGATCTCTGTACGGRAGGCAGCA G-3, and reverse: 5-CGCTCTTCCGATCTGACGGACTACHVGGGTWTCTAAT-3. 16S rRNA gene amplicon sequencing was performed according to a previously described protocol [38]. 10,000 reads per sample for NIBIOHN cohort and 5,000 reads per sample for MORINAGA cohort were randomly selected for further analysis. Samples with insufficient read numbers were resequenced, and samples with repeated insufficient read numbers were thereafter excluded.

### Bioinformatics analysis

The obtained paired end FASTQ data were trimmed and merged before selection of the operational taxonomic units (OTUs). The OTU classification and diversity analysis were performed using QIIME pipeline (v1.9.1) [39]. All the steps from FASTQ trimming to gut microbiota diversity analysis were automatically performed according to a previously described method [40]. The OTUs were clustered against SILVA 128 reference database [41] at 97% similarity using USEARCH algorithm [42]. Taxonomic classification was performed using SILVA 128 reference database to the genus level taxa (hereafter referred as genera). Taxonomy name is expressed with a specific taxonomy name based on the SILVA database phylogenetic classification standard (https://www.arb-silva.de/browser/ssu/).

### Statistical analysis

The output of QIIME pipeline in Biom table format was imported and analyzed in R (version 3.5.1). The alpha-diversity indices were calculated by the *estimate_richness* function in the phyloseq R-package. For alpha-diversity comparison analysis between NIBIOHN and MORINAGA cohorts, 5000 reads were randomly reselected from NIBIOHN cohort. The beta-diversity index, calculated by Bray-Curtis distance using genus level data, was generated using the *vegdist* function in the vegan R-package. For enterotype analysis, we used Jensen-Shannon divergence (JSD) using a previously described method [6]. Principal coordinate analysis (PCoA) was performed using the *dudi.pco* function in the ade4 R-package. Covariates of gut microbiome -diversity were identified by calculating the association between continuous or categorical phenotypes and genus-level community coordinate with *envfit* function in the vegan R-package. This function performs MANOVA and linear correlations for categorical and continuous variables, respectively. Over all metadata categories, 68 covariates in the NIBIOHN cohort and 32 covariates in the MORINAGA cohort were identified. To identify non-redundant determinants of microbiota variation, the covariates selected by *envfit* function were sub-selected by forward stepwise redundancy analysis on genus-level community ordination calculated by Bray-Curtis distance with the *ordiR2step* function in the vegan R-package. The dominant bacteria from phylum to genus level were defined as the mean of the distribution of bacterial composition with at least 1% correlation analysis. We used the Wilcoxon rank sum test (*wilcox.test* function in stats R-package) and Spearman correlation analysis (*cor* function in stats R-package) for comparison and correlation analysis, respectively. The comparison analysis of metadata was based on summary statistics of data derived from each cohort. Heatmaps were created using corrplot and superheat R-package, and PCoA figures and Boxplots were created using R package ggplot2. All statistical tests were two-sided, with a *p*-value <0.05 considered significant. The package information and function information used in this study were released through github (https://github.com/Jonguk-microbiome/Japanese_gut_microbiome_analysis/blob/main/Analysis_R_script.R).

### Data availability

The DNA sequences corresponding to the 16S rRNA gene for NIBIOHN cohort have been deposited in DNA Databank of Japan (DDBJ) under accession numbers DRA010837 DRA010841 (https://ddbj.nig.ac.jp/DRASearch/study?acc=DRP007218, DRP007219, DRP007220, DRP007221 DRP007222) and the DNA sequences for MORINAGA cohort have been deposited in DDBJ under accession numbers DRA009764 DRA009767 (https://ddbj.nig.ac.jp/DRASearch/study?acc=DRP005906).

## Results

### Distribution of gut bacterial community in healthy individuals of two independent cohorts

**Fig. 1:**
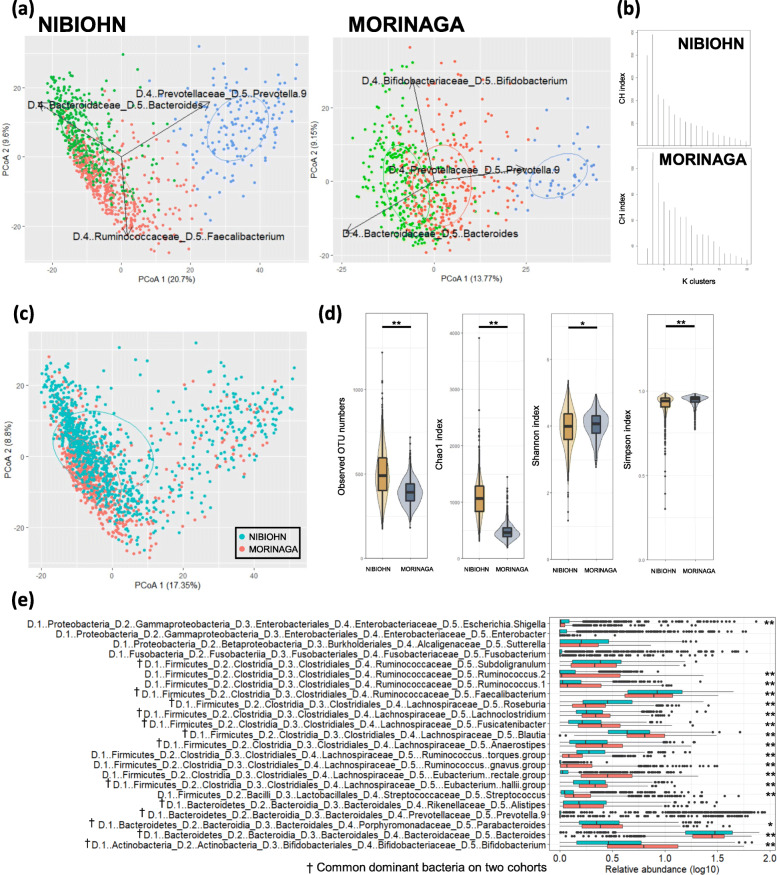
Gut microbiota distribution of the NIBIOHN cohort and MORINAGA cohort (**a**) Microbial community variation in each cohort represented by principal coordinates analysis (PCoA, genus-level JSD) and PAM clustering. Arrows indicated enterotype drivers; *Bacteroides*-enterotype (green), *Prevotella*-enterotype (blue), and *Faecalibacterium*-enterotype or *Bifidobacterium*-enterotype (red). **b** The estimated result of suitability of cluster number Calinski-Harabasz index (CH index) (**c**) The distribution of integrated data of two cohorts (**d**) The distribution of alpha diversity indices of NIBIOHN cohort (yellow) and MORINAGA cohort (blue) (**e**) The dominant genus among the two cohorts and their composition. **p<0.01, *p<0.05 (Wilcoxon rank sum test)

First, we described the gut bacterial community structure of two large-scale healthy Japanese cohorts based on genus-level PCoA and enterotype analysis by partitioning around medoids (PAM) clustering using JSD (Fig.1a and Fig. S2a, b). Values of the Calinski-Harabasz index (CH index) suggested that each cohort was divided into three clusters (Fig. 1b), which were characterized by the predominance of *Bacteroides, Prevotella_9,* and *Faecalibacterium* (in the NIBIOHN cohort) or *Bifidobacterium* (in the MORINAGA cohort). The ratio of each enterotype were 37:16:47 (*Bacteroides*-enterotype: *Prevotella*-enterotype: *Faecalibacterium*-enterotype) in the NIBIOHN cohort and 50:8:42 (*Bacteroides*-enterotype: *Prevotella*-enterotype: *Bifidobacterium*-enterotype) in the MORINAGA cohort. These results indicated that *Faecalibacterium* in the NIBIOHN cohort and *Bifidobacterium* in the MORINAGA cohort largely contributed to the individual differences in characteristics and distribution of the gut bacterial community. PCoA based on the integrated data from the two cohorts showed that no obvious difference in overall distribution between the two cohorts was observed (Fig. 1c). We also integrated the two cohorts and analyzed the enterotype. The integrated data of Japanese gut microbiota could be divided into two enterotypes based on the CH-index (Fig. S1d), *Bacteroides*-enterotype and *Prevotella*-enterotype (Fig. S2c). The integrated data also showed that the other enterotype-related genera of each cohort, *Faecalibacterium* and *Bifidobacterium*, exhibited similar directionalities on gut microbial variation (Fig. S2c). The comparison of the alpha diversity indices showed differences between the two cohorts; NIBIOHN cohort contained a significantly higher number of observed OTUs and species richness Chao1 related to alpha diversity index (*p*-value <0.01), whereas MORINAGA cohort was significantly higher in the Shannon and Simpson diversity index related to alpha diversity evenness (p-value <0.05 and p-value <0.01, respectively) (Fig. 1d). Next, we compared the predominant genus of gut microbiota in the two cohorts. Thirteen genera including *Alistipes, Anaerostipes*, *Bacteroides, Bifidobacterium, Blautia*, *Eubacterium halli* group, *Faecalibacterium*, *Fusicatenibacter*, *Lachnoclostridium*, *Parabacteroides*, *Prevotella_9*, *Roseburia* and *Subdoligranulum* were the predominant bacterial genera and were common among the two cohorts (Fig. 1e). The distribution of these genera was slightly different between the two cohorts. NIBIOHN cohort showed a significant abundance of *Bacteroides*, *Faecalibacterium*, and *Roseburia,* and MORINAGA cohort showed an abundance of *Bifidobacterium*, *Blautia*, *Lachnoclostridium*, *Fusicatenibacter*.

### Identification of metadata covarying with gut microbiota in Japanese populations

**Fig. 2:**
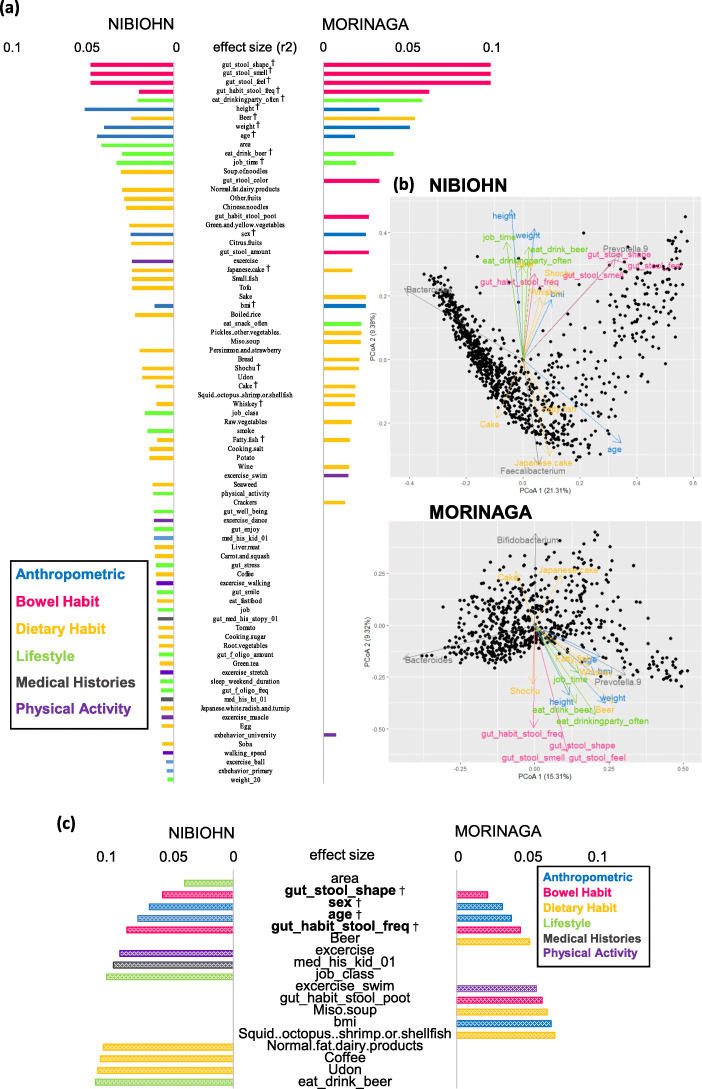
Effect size of microbiome covariates (**a**) Effect size identified in the NIBOHN cohort (left) and the MORINAGA cohort (right). Factors are sorted according to their effect size and colored based on metadata category (Table S1) (**b**) PCoA-based on Bray-Curtis distance. Arrows show the ordination of 18 common covariates for overall microbiome community variation in the NIBIOHN cohort (top) and the MORINAGA cohort (bottom) (**c**) Cumulative effect size of non-redundant covariates. Microbial covariates selected by stepwise redundancy analysis in the NIBIOHN cohort (left) and the MORINAGA cohort (right). Common covariates in the two cohorts

Our results highlighted the common determinants that lead to variations in gut microbiota in the Japanese population. We tested a total of 134 variables to identify covariates associated with gut bacteria (Table S1). The comparison of metadata among the two cohorts indicated there were certain cohort-specific features (Table S2), for example, intake of dairy products and area of residence (Table S2 and Fig. S1). Univariate analysis, using the *envfit* function, for overall variations in the microbiome revealed 18 matching variables to be significantly associated with microbiome composition in both cohorts (*p*-value <0.05, Fig.2a, Table S3). A majority of the matching variables indicated a comparatively high effect size in each cohort. In particular, bowel habit-related variables showed strong associations with gut microbiota. Five anthropometric variables such as height, weight, age, gender, and BMI, were also common between the two cohorts. Although several diet-related variables were particularly significant, alcoholic drinks (sake, beer, shochu, whiskey, and wine), sweets (cake and Japanese cake), and fatty fish were common in the two cohorts. In addition, a high consumption of beer, among all alcoholic drinks, (Table S2) was also common. In the lifestyle category, the frequency of social drinking and total weekly working hours were common in the two cohorts. There were no significant common variables in the medical history or physical activity category. Some variables also showed similar directionalities for the ordination of gut microbiota composition in the two cohorts, for example, stool odor, BSS, and a feeling of exhilaration during bowel movement, and frequency of bowel movement per week (Fig. 2b).

**Fig. 3:**
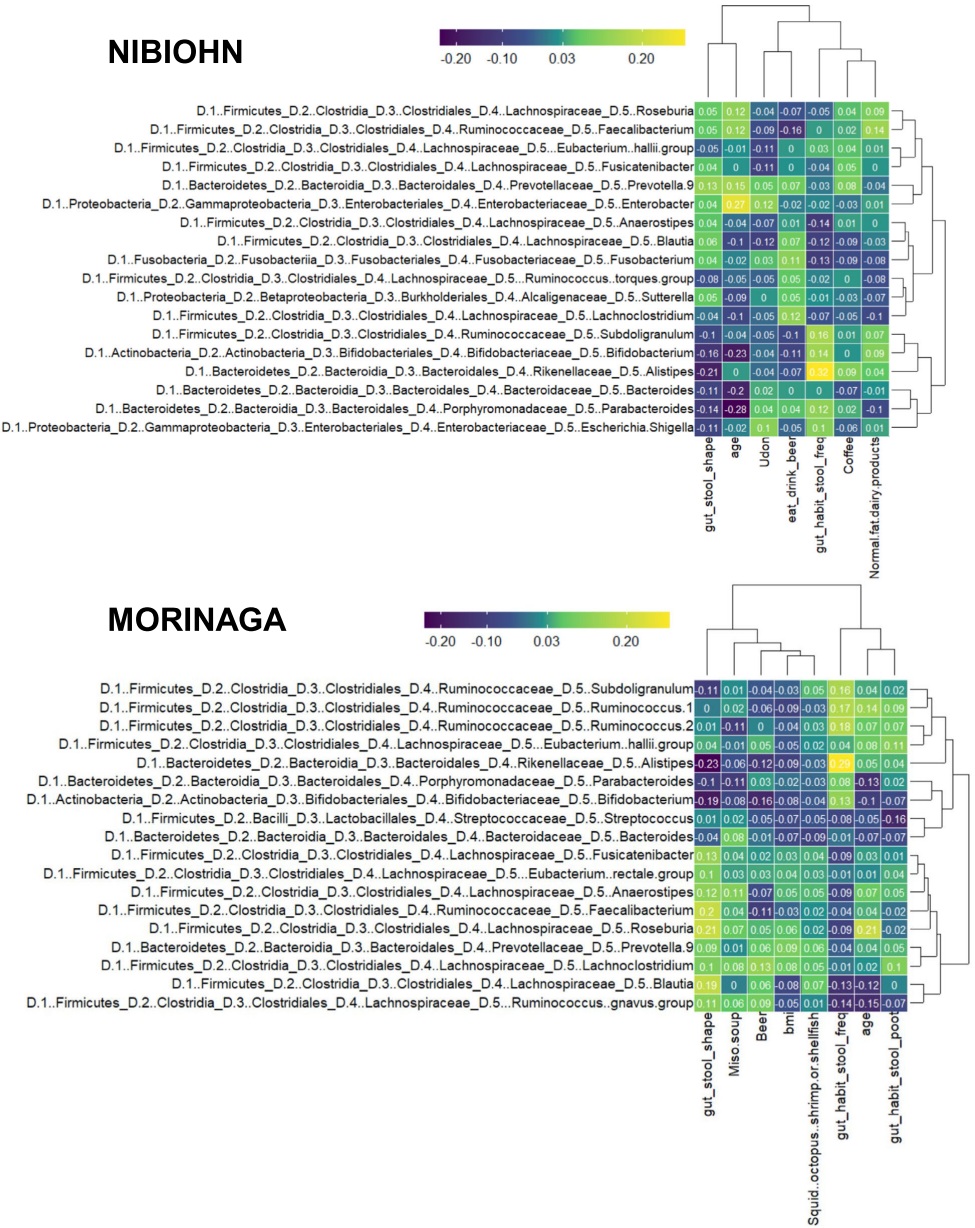
Correlation matrix heatmap between dominant bacteria and non-redundant covariates selected by stepwise redundancy analysis, as calculated by Spearmans rank correlation coefficients, in NIBIOHN cohort (top) and MORINAGA cohort (bottom)

We observed a redundancy among covariates as there was an overlap in the directionalities of variables (Fig. 2b and c). To identify non-redundant covariates of microbiome variations, we performed a forward stepwise redundancy analysis (vegan: *ordiR2 step* function). The number of non-redundant covariates were 12 out of 68 variables in the NIBIOHN cohort, and 10 out of 32 variables in the MORINAGA cohort, and their cumulative effect size on microbial community variation were 10.7% in the NIBIOHN cohort and 7.1% in the MORINAGA cohort, respectively. Furthermore, we demonstrated that four covariates, BSS, gender, age, and frequency of bowel movement per week, matched in the two cohorts (Fig. 2c). In addition, although the proposed question in the questionnaire was different in the two cohorts, factors related to beer consumption were identified as non-redundant factors. The other non-redundant covariates showed diverse characteristic features in each cohort. In particular, the residential area of participants had no significant influence on the gut microbial variance in the MORINAGA cohort, but showed the highest effect size on gut microbial variance in the NIBIOHN cohort (*p*-value <0.001). Furthermore, the Spearman correlation analysis based on the dominant-genus data indicated that there were corresponding associations between these four factors (BSS, gender, age, and frequency of bowel movement) and gut bacteria (Fig.3 and Table S4), as well as overall microbiome community variations. BSS showed inverse correlation with *Bifidobacterium* and *Alistipes*. The differences between genders were illustrated by high prevalence of *Prevotella_9* in men, and *Alistipes, Bifidobacterium, Faecalibacterium* and *Subdoligranulum* in women. The relative abundance of *Roseburia* increased with age, whereas that of *Blautia* and *Parabacteroides* decreased with age. Bowel movement frequencies per week were positively associated with the abundance of *Subdoligranulum* and *Alistipes*, and negatively associated with the presence of *Blautia*.

## Discussion

In the present study, we report that not only the predominant bacterial genera (Fig. 1e), but also their interactions between metadata covariates were common (Fig. 2c) in two independent cohorts and seemed to show the general characteristics of healthy gut microbiota in the Japanese populations. First, we investigated the overall structural characteristics. Although one of the dominant genera, *Faecalibacterium* in the NIBIOHN cohort and *Bifidobacterium* in the MORINAGA cohort, seemed to be different between the two cohorts (Fig. 1a, b), integrated data indicated that these genera had a similar third directionality on gut microbial variations (Fig. S2c). Because the directionalities of the two genera did not exactly correspond, the third directionality was not chosen as an enterotype. Taken together with a previous report analyzing the relationship between enterotypes and assemblages [43], the gut microbiota could be divided into three clusters.

To clarify the features of gut microbiota in Japanese populations in detail, we focused on the dominant genus in each cohort, and showed 13 genera were common in the two cohorts (Fig. 1e). Compared with the dominant genera in a dataset containing a total of 2186 North American and European individuals [44], most of our dominant genera were common, but the average relative abundance varied. The characteristic differences in abundance were that of *Bifidobacterium,* which was 4.4% in the NIBIOHN cohort, 8.4% in the MORINAGA cohort, as compared to 1.4% in the previous report [44], and that of *Blautia*, which was 4.7% in the NIBIOHN cohort, 6.5% in the MORINAGA cohort (Fig. 1e), and 2.9% in the previous report [44]. Altogether, the dominant genera seemed to be common regardless of the population, however, the abundance of genera showed population-specific features.

There were some cohort-specific features in gut bacteria (Fig. 1c) and alpha diversity indices (Fig. 1d) in the two cohorts, even though they both comprised of Japanese populations. It was suggested that these differences between the two cohorts were due to subjects having different backgrounds as indicated in Table S2. Together with a previous study [38], which reported that consumption of dairy products increases *Bifidobacterium* abundance in Japanese population, the higher intake of normal fat dairy products in the MORINAGA cohort compared to the NIBIOHN cohort (Table S2) was thought to be one of the reasons for a significant difference in *Bifidobacterium* abundance. Most of the subjects in the MORINAGA cohorts were customers who purchased products from the Morinaga Milk Industry Co., Ltd.

The comparison of results by *envfit* analysis between the two Japanese cohorts revealed 18 common covariates, showing significant association with structural variations in microbiota (Fig. 2a). Seven common covariates including BSS, height, weight, BMI, alcohol consumption (beer, rice wine, and low alcohol liquor), age, and gender, were in agreement with previous reports of three different cohorts in Belgium [29], Netherland [30], and China [31]. It appears that regardless of the population, these seven covariates commonly associate with structural differences in gut microbiota. Significant common covariates involved in microbial variation in the three cohorts [29,31] of previous studies, such as smoking, disease state, medication, fruit and meat consumption, were not observed in the NIBIOHN and MORINAGA cohorts, while the significant association of cake and fatty fish consumption was not found in previous cohorts [29,31]. These differences may be attributed to distinctive dietary habits between populations. For instance, Japanese population show the highest intake of seafood in the world [45], which may be a contributing factor to gut microbial variations. Although Falony et al. [29] revealed that medication resulted in the largest total variance and interacted with other covariate-microbiota associations, the target of our study was different and the effects of medication showed no significant association with healthy gut microbiota.

A forward stepwise redundancy analysis showed a remarkable cumulative effect size on community variation, specifically 10.7% in the NIBIOHN cohort and 7.1% in the MORINAGA cohort (Fig. 2c); effect size in both Japanese cohorts were comparable to 7.7% reported in Belgian population [29]. These results indicated that the proportion of gut microbial variation explained by questionnaire-based covariates seemed to be approximately 10% regardless of the population or number of covariates, suggesting there were several additional intrinsic or extrinsic contributors such as immunity, host genetics, bacterial-bacterial interaction, as well as unknown factors [46]. Non-redundant determinants such as BSS, gender, and age were common in our two cohorts (Fig. 3c), in accordance with the previous report involving a Belgian cohort [29], indicating that these covariates are common among healthy populations independent of factors like country of origin. Falony et al. [29] reported that stool consistency showed the largest effect size on bacterial variations; BSS score for gut_stool_shape showed a higher non-redundant effect on total composition variation in Japanese cohorts (Fig. 2c). Gender [15] and age [9] intricately associated with life style factors such as dietary habits, therefore, the selection of gender and age as non-redundant covariates was justified. Interestingly, non-redundant analysis also showed population- or cohort-specific results. Beer consumption and related covariates were identified as non-redundant covariates in each cohort (Fig. 2c), presumably because beer was the most popular alcoholic drink in the Japanese cohorts (Table S2). This result is in line with a previous study [47] stating that alcohol affects the composition of gut bacteria.

Another cohort-specific feature with a high effect size was observed to be residential area in the NIBIOHN cohort, whereas no association was observed in the MORINAGA cohort (Table S3). The distribution of residential area was extremely different between the two cohorts; NIBIOHN cohort comprised of individuals from a limited area with a high concentration of subjects in each area (Fig. S2), and MORINAGA cohort comprised of individuals from a dispersed and continuous area. Notably, a previous report, involving a 16S rRNA gene analysis of fecal samples collected from 516 healthy Japanese adults residing in various regions of Japan [33], was very similar to the MORINAGA cohort, and demonstrated no association between residential area and gut microbiota variation. However, the residential area was found to have the highest influence on gut bacterial variance in the NIBIOHN cohort. Further large-scale nationwide cohort studies are required to understand the effect of residential area on total gut microbial variance among Japanese populations.

Besides the common interactions of covariates and compositional variation, some associations between gut bacteria and covariates were also common between the two cohorts (Fig. 3 and Table S4). The negative association of BSS score with *Alistipes* was in accordance with a previous report [29], whereas the negative association with *Bifidobacterium* was not previously reported. Furthermore, the reported association of Christensenellaceae, Mogibacteriaceae, and Rikenellaceae with bowel movement frequency in the Japanese population [33] was not observed in our study. Interestingly, regardless of the influence of BSS score and frequency of bowel movement on colon transit time, both covariates showed non-redundant associations with inter-individual gut microbial variation. This difference was represented by the fact that *Subdoligranulum* and *Blautia* only associated with defecation frequency. This difference highlighted the complex association between colon transit time, gut microbiota, and diet [27]. In relation to the common association of gender and gut bacteria, the higher abundance of *Bifidobacterium* in women and *Prevotella_9* in men (Table S4) was in agreement with a previous report of Japanese gut microbiota [34], indicating the possibility of population-specific results. In contrast, the higher abundance of *Faecalibacterium* and *Alistipes* in women (Table S4) is first reported in the present study. The common association of age with the abundance of *Blautia*, *Parabacteroides,* and *Roseburia* was not shown in the previous report [29] and seemed to be population-specific. A decrease of *Bifidobacterium* abundance with age in the NIBIOHN cohort was in accordance with previous reports [8, 29], identifying *Bifidobacterium* as an adult-enriched bacteria. However, in the MORINAGA cohort, *Bifidobacterium* showed no significant association with age. One of the reasons for this discrepancy was presumed to be the unique dietary habits, which was high consumption of dairy products, and the high relative abundance of *Bifidobacterium* in the MORINAGA cohort.

In this study, we reported the characteristics of gut microbiota and comprehensively examined the major microbiome-associated variables in Japanese gut microbiota using two independent large-scale cohort data. The comparison between these large, distinct cohorts provided reliability and robustness to our study. We were unable to analyze the data under exactly same conditions due to the circumstances of each institution, logistics, time, etc. We analyzed the results for each cohort considering the batch effect between the two large cohorts and compared the results, but we do not understand the effect on each result. Our study was a cross-sectional study and did not show the causal relationships between gut microbiota and metadata. Prospective observational or interventional studies would be required to delineate these relationships. The limitation of this study was that all participants were applicants, which may be a source of potential bias in this study. Random sampling of subjects from all over the country or a larger sample size would be essential for overcoming this bias.

## Conclusions

Our results described the features of healthy Japanese gut microbiota, including 13 predominant genera and high abundance of *Bifidobacterium* and *Blautia,* in two independent cohorts. The comparison of two diverse and independent cohorts increased the reliability of our results. We found that despite the presence of a batch effector between the two large cohorts, a common covariate affects the gut microbial community. 18 covariates, including anthropometric measurements, bowel habits, lifestyle, and dietary habits, commonly associated with gut bacterial variations were identified. Furthermore, the BSS score, gender, age, and frequency of bowel movement independently affected gut microbiota composition and were inferred to be essential factors influencing microbial communities in healthy Japanese populations. To our knowledge, this was the first study to report integrated and non-redundant associations of factors affecting structural characteristics of gut microbiota among Japanese populations.

## Supplementary Information

**Additional file 1: TableS1**. The information of phenotype data using this study.

**Additional file 2: TableS2**. Comparison of phenotype data between two cohorts.

**Additional file 3: TableS3**. Univariate analysis for overall microbiome community variation using the envfit function.

**Additional file 4: TableS4**. Correlation analysis of selected four phenotype factors after non-redundant covariates analysis and dominant gut bacteria (FDR<0.05).

**Additional file 5: Figure S1.** The distribution of area in two cohorts (left: NIBIOHN cohort, right: MORINAGA cohort).

**Additional file 6: Figure S2.** Gut microbiota distribution of NIBIOHN cohort (a), MORINAGA cohort (b) and the merged data of the two cohorts (c) by principal coordinates analysis (PCoA, genus-level JSD) and PAM clustering. Arrows indicated the ordination of dominant genus. (d) The estimated result of suitability of cluster number Calinski-Harabasz Index (CH index) on the two cohorts-merged data.

**Additional file 7.**Genus-level taxonomy relative abundance information.

## Data Availability

The DNA sequences corresponding to the 16S rRNA gene for NIBIOHN cohort have been deposited in DDBJ under accession numbers DRA010837 DRA010841 (https://ddbj.nig.ac.jp/DRASearch/study?acc=DRP007218, DRP007219, DRP007220, DRP007221, DRP007222), and the sequences for MORINAGA cohort have been deposited in DDBJ under accession numbers DRA009764DRA009767 (https://ddbj.nig.ac.jp/DRASearch/study?acc=DRP005906). Taxonomy relative abundance information analyzed by pipeline in this study is provided in Additionalfile7. The datasets supporting the results of this study are included within the article and Additional files. The package information and function information used in this study were released through github (https://github.com/Jonguk-microbiome/Japanese_gut_microbiome_analysis/blob/main/Analysis_R_script.R).

## Abbreviations

NIBIOHN: National Institutes of Biomedical Innovation, Health and Nutrition
BSS: Bristol Stool Scale
PAM: Partitioning around medoids
BMI: Body mass index
BDHQ: Brief Self-Administered Diet History Questionnaire
OTU: Operational taxonomic unit
CH index: Calinski-Harabasz index
JSD: Jensen-Shannon divergence
PCoA: Principal coordinate analysis
