# Supplementary material for: Comprehensive analysis of gut microbiota of a healthy population and covariates affecting microbial variation in two large Japanese cohorts
Source: BMC Microbiol. 2021 May 20;21:151. doi: 10.1186/s12866-021-02215-0 (PMC8139087; doi:10.1186/s12866-021-02215-0)
Supplement: Supplementary file 5 — Additional file 5: Figure S1. The distribution of area in two cohorts (left: NIBIOHN cohort, right: MORINAGA cohort). [file 12866_2021_2215_MOESM5_ESM.pptx]

## Slide 1
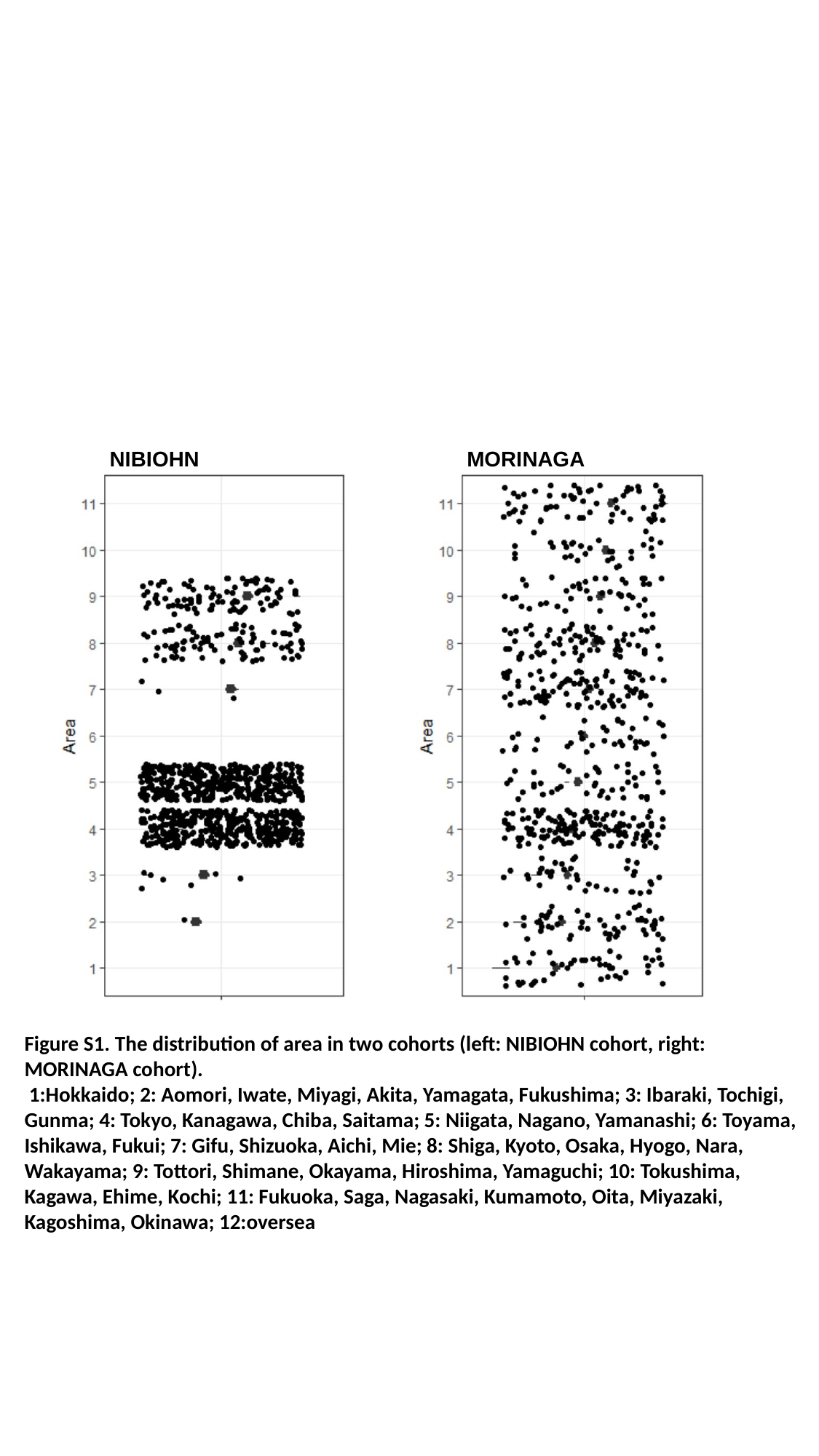

NIBIOHN
MORINAGA
Figure S1. The distribution of area in two cohorts (left: NIBIOHN cohort, right: MORINAGA cohort).
 1:Hokkaido; 2: Aomori, Iwate, Miyagi, Akita, Yamagata, Fukushima; 3: Ibaraki, Tochigi, Gunma; 4: Tokyo, Kanagawa, Chiba, Saitama; 5: Niigata, Nagano, Yamanashi; 6: Toyama, Ishikawa, Fukui; 7: Gifu, Shizuoka, Aichi, Mie; 8: Shiga, Kyoto, Osaka, Hyogo, Nara, Wakayama; 9: Tottori, Shimane, Okayama, Hiroshima, Yamaguchi; 10: Tokushima, Kagawa, Ehime, Kochi; 11: Fukuoka, Saga, Nagasaki, Kumamoto, Oita, Miyazaki, Kagoshima, Okinawa; 12:oversea
