# Supplementary material for: Comprehensive analysis of gut microbiota of a healthy population and covariates affecting microbial variation in two large Japanese cohorts
Source: BMC Microbiol. 2021 May 20;21:151. doi: 10.1186/s12866-021-02215-0 (PMC8139087; doi:10.1186/s12866-021-02215-0)
Supplement: Supplementary file 6 — Additional file 6: Figure S2. Gut microbiota distribution of NIBIOHN cohort (a), MORINAGA cohort (b) and the merged data of the two cohorts (c) by principal coordinates analysis (PCoA, genus-level JSD) and PAM clustering. Arrows indicated the ordination of dominant genus. (d) The estimated result of suitability of cluster number Calinski-Harabasz Index (CH index) on the two cohorts-merged data. [file 12866_2021_2215_MOESM6_ESM.pptx]

## Slide 1
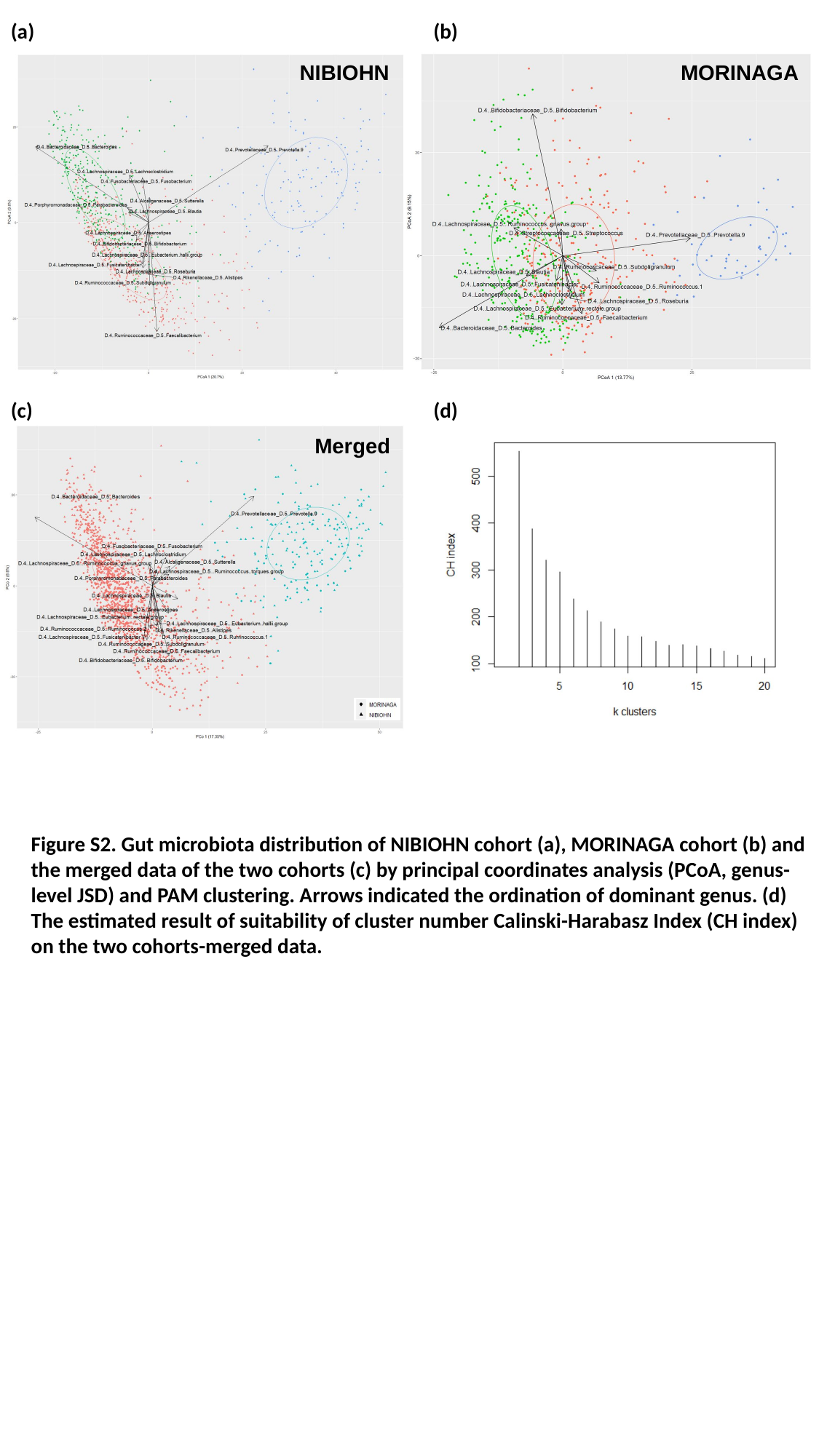

(a)
(b)
NIBIOHN
MORINAGA
(c)
(d)
Merged
Figure S2. Gut microbiota distribution of NIBIOHN cohort (a), MORINAGA cohort (b) and the merged data of the two cohorts (c) by principal coordinates analysis (PCoA, genus-level JSD) and PAM clustering. Arrows indicated the ordination of dominant genus. (d) The estimated result of suitability of cluster number Calinski-Harabasz Index (CH index) on the two cohorts-merged data.
